# Serum autophagy-related gene 5 level in stroke patients: correlation with CD4^+^ T cells and cognition impairment during a 3-year follow-up

**DOI:** 10.1590/1414-431X2024e13019

**Published:** 2024-03-18

**Authors:** Juanjuan Qu, Linxia Wu, Meng Zhang, Minchen Kan, Huimin Chen, Yanqing Shi, Shuangyu Wang, Xiaohua Wang, Fan Chen

**Affiliations:** 1 Department of Emergency, Handan Central Hospital, Handan, China; 2 Department of Nephrology, Handan Central Hospital, Handan, China; 3 Department of Neonatal Ward, Handan Central Hospital, Handan, China

**Keywords:** Stroke, Autophagy-related gene 5, Th1/Th2 cells, Th17/Treg cells, Cognition impairment

## Abstract

Autophagy-related gene (ATG) 5 regulates blood lipids, chronic inflammation, CD4^+^ T-cell differentiation, and neuronal death and is involved in post-stroke cognitive impairment. This study aimed to explore the correlation of serum ATG5 with CD4^+^ T cells and cognition impairment in stroke patients. Peripheral blood was collected from 180 stroke patients for serum ATG5 and T helper (Th) 1, Th2, Th17, and regulatory T (Treg) cell detection via enzyme-linked immunosorbent assays and flow cytometry. The Mini-Mental State Examination (MMSE) scale was completed at enrollment, year (Y)1, Y2, and Y3 in stroke patients. Serum ATG5 was also measured in 50 healthy controls (HCs). Serum ATG5 was elevated in stroke patients compared to HCs (P<0.001) and was positively correlated to Th2 cells (P=0.022), Th17 cells (P<0.001), and Th17/Treg ratio (P<0.001) in stroke patients but not correlated with Th1 cells, Th1/Th2 ratio, or Treg cells (all P>0.050). Serum ATG5 (P=0.037), Th1 cells (P=0.022), Th17 cells (P=0.002), and Th17/Treg ratio (P=0.018) were elevated in stroke patients with MMSE score-identified cognition impairment *vs* those without cognition impairment, whereas Th2 cells, Th1/Th2 ratio, and Treg cells were not different between them (all P>0.050). Importantly, serum ATG5 was negatively linked with MMSE score at enrollment (P=0.004), Y1 (P=0.002), Y2 (P=0.014), and Y3 (P=0.001); moreover, it was positively related to 2-year (P=0.024) and 3-year (P=0.012) MMSE score decline in stroke patients. Serum ATG5 was positively correlated with Th2 and Th17 cells and estimated cognitive function decline in stroke patients.

## Introduction

Stroke, a common cerebrovascular disease, is composed of ischemic and hemorrhagic subtypes (1). In 2019, there were 12.2 million incident strokes and 101 million prevalent strokes, of which approximately 62.4% were ischemic stroke (2). Along with the disease progression, vessel occlusion or rupture leads to structural and functional changes in brain tissue, leading to excitotoxicity, oxidative stress, blood-brain barrier dysfunction, inflammation, and neural cell death (3). The aforementioned processes can result in irreversible brain damage, leading to cognition impairment (4). Furthermore, patients with cognition impairment following stroke often experience quality-of-life issues, long-term care support, and mental burden (5). Even worse, post-stroke cognition impairment is implicated in the development of vascular dementia, making stroke a leading cause of the disability epidemic in adults (6). Thus, it is necessary to search for associated molecular biomarkers to monitor, predict, and intervene in the incidence and deterioration of cognition impairment to improve stroke management (6,7).

Autophagy-related gene (ATG) 5, as a critical autophagy regulator, is impacted by the modification of CD4^+^ T-cell differentiation, atherosclerosis, and neuronal damage (8-16). For instance, one study shows that ATG5 regulates T-helper (Th) 1, Th17, and T regulatory (Treg) cells in asthma mice (9). In addition, another study shows that mice with a macrophage-specific deletion of ATG5 are more likely to develop atherosclerotic plaques (11). Furthermore, knockdown of ATG5 attenuates ischemia-reperfusion injury (IRI) in an experimental ischemic stroke mouse model (12). Several other studies elucidate that ATG5 can regulate neuronal apoptosis, further participating in neuronal damage (13-15). Regarding the clinical implication of ATG5 in stroke, only one previous study shows that the serum level of ATG5 can be used to identify patients with early-stage ischemic stroke (16). However, the role of serum ATG5 level in estimating cognition impairment in stroke patients is unclear.

Hence, this study quantified serum ATG5 level and the CD4^+^ T-cell subset, aiming to investigate their inner correlation and their linkage with cognition impairment incidence and deterioration in stroke patients.

## Material and Methods

### Subjects

A total of 180 consecutive stroke patients who visited or were followed up at Handan Central Hospital between April 2019 and February 2020 were included. The inclusion criteria were as follows: a) confirmed stroke via the guidelines from the American Heart Association/American Stroke Association (AHA/ASA) (17); b) older than 18 years of age; and c) no intracranial hemorrhage. The exclusion criteria were as follows: a) complications with malignancies or immune system diseases; b) concomitant with global or receptive aphasia; c) active infection; and d) pregnancy or breastfeeding. In addition, fifty subjects were enrolled as healthy controls (HC). The eligible criteria were as follows: a) no abnormalities in recent physical examinations; b) age- and sex-matched stroke patients; c) no malignancies or immune system diseases; d) no stroke history or subclinical stroke; e) no active infections; and f) not pregnant or breastfeeding. The Ethics Committee of Handan Central Hospital approved the study, and subjects or guardians signed a written informed consent.

### Collection and detection

Demographic information, chronic comorbidities, and disease information of stroke patients were recorded in detail. The age and sex of the healthy controls were documented. Peripheral blood (PB) was collected from all subjects at enrollment. Following that, the PB was divided into two parts: a) one part was used to isolate serum and detect ATG5 levels by enzyme-linked immunosorbent assays (ELISA) using Human ATG5 ELISA kits (Clone Cloud, USA); and b) one part was used to quantify the CD4^+^ T cells (T helper (Th) 1, Th2, Th17, and regulatory T (Treg)) by flow cytometry using the Human Th1/Th2 Phenotyping Kit and Human Th17/Treg Phenotyping Kit (BD, USA). The experiments strictly followed the kit instructions in triplicate.

### Assessment

After enrollment, stroke patients were evaluated by the mini-mental state examination (MMSE), and then the cognition impairment (MMSE score <27) was assessed (18). After admission, routine follow-up was conducted in stroke patients for 3 years, during which the MMSE score was recorded at year 1, year 2, and year 3. Sequentially, the 1-year, 2-year, and 3-year MMSE score declines were calculated, which were defined as the MMSE score at enrollment minus the MMSE score at year 1, year 2, or year 3. Due to loss to follow-up, the missing data were not included in the analysis.

### Statistical analysis

SPSS v.22.0 (IBM, Inc., USA) and GraphPad Prism v.7.0 (GraphPad Software, Inc., USA) were utilized for analyses and plotting, respectively. Data were compared using the Mann-Whitney U test. Correlations were evaluated using the Spearman test. No adjustment for covariates and demographic information was performed. P<0.05 was considered significant.

## Results

### Clinical characteristics of stroke patients and healthy controls

The 180 stroke patients had a mean age of 68.1±7.8 years; 66 (36.7%) were female and 114 (63.3%) were male. The median Th1/Th2 and Th17/Treg ratios were 1.3 (0.9-1.9) and 0.7 (0.5-1.2), respectively. The mean MMSE score was 26.4±2.0. Seventy-nine (43.9%) stroke patients had cognition impairment. In addition, the mean age of healthy controls was 63.9±7.3 years. There were 20 (40%) females and 30 (60%) males in the healthy control group. The detailed clinical information (including demographic characteristics, disease history, and disease features) is listed in Table 1.

**Table 1:** Clinical characteristics of stroke patients and healthy controls.

Items	Stroke patients (n=180)	Healthy controls (n=50)
Age (years), mean±SD	68.1±7.8	63.9±7.3
Gender, n (%)		
Female	66 (36.7)	20 (40.0)
Male	114 (63.3)	30 (60.0)
BMI (kg/m^2^), mean±SD	24.9±2.4	N/A
Smoking history, n (%)	89 (49.4)	14 (28.0)
Drinking history, n (%)	73 (40.6)	12 (24.0)
Education level, n (%)		
Primary school or below	57 (31.7)	11 (22.0)
Middle or high school	87 (48.3)	26 (52.0)
Undergraduate or above	36 (20.0)	13 (26.0)
Marital status, n (%)		
Married	97 (53.9)	34 (68.0)
Single/divorced/widowed	83 (46.1)	16 (32.0)
Household register, n (%)		
Rural	15 (8.3)	6 (12.0)
Urban	165 (91.7)	44 (88.0)
Hypertension history, n (%)	146 (81.1)	0 (0.0)
Hyperlipidemia history, n (%)	88 (48.9)	0 (0.0)
Diabetes history, n (%)	54 (30.0)	0 (0.0)
CKD history, n (%)	53 (29.4)	0 (0.0)
CVD history, n (%)	73 (40.6)	0 (0.0)
Lesion location, n (%)		
Left	72 (40.0)	N/A
Right	70 (38.9)	N/A
Bilateral/brainstem/unknown	38 (21.1)	N/A
Recurrent experience of stroke, n (%)		
No	131 (72.8)	N/A
Yes	49 (27.2)	N/A
Th1 cells (%), median (IQR)	15.6 (12.8-20.8)	N/A
Th2 cells (%), median (IQR)	11.9 (9.6-16.2)	N/A
Th1/Th2 ratio, median (IQR)	1.3 (0.9-1.9)	N/A
Th17 cells (%), median (IQR)	3.9 (2.9-5.5)	N/A
Treg cells (%), median (IQR)	5.0 (4.1-7.0)	N/A
Th17/Treg ratio, median (IQR)	0.7 (0.5-1.2)	N/A
MMSE score, mean±SD	26.4±2.0	N/A
Cognition impairment, n (%)	79 (43.9)	N/A

SD: standard deviation; BMI: body mass index; CKD: chronic kidney disease; CVD: cardiovascular disease; Th: T helper; Treg: regulatory T; MMSE: mini-mental state examination. Mann-Whitney U test, *t*-test, or chi-squared test. N/A: not available.

### Comparison of serum ATG5 level between stroke patients and healthy controls

**Figure 1:**
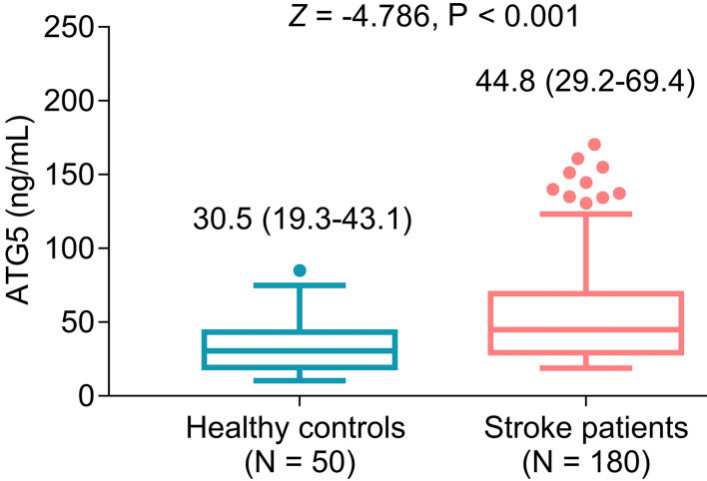
Serum autophagy-related gene (ATG) 5 level was elevated in stroke patients compared to healthy controls. Data are reported as median and interquartile range. Mann-Whitney U test.

Serum ATG5 level was elevated in stroke patients compared to healthy controls (P<0.001). In detail, the median and interquartile range (IQR) of serum ATG5 level were 44.8 (29.2-69.4) ng/mL in stroke patients and 30.5 (19.3-43.1) ng/mL in healthy controls (Figure 1).

### Comparison of CD4^+^ T-cell subset between female and male stroke patients

No difference was found in Th1 cells (P=0.838), Th2 cells (P=0.641), or Th1/Th2 ratio (P=0.740) between female and male stroke patients. Th17 cells (P=0.565), Treg cells (P=0.304), and Th17/Treg ratio (P=0.964) also did not vary between female and male patients (Supplementary Table S1).

### Correlation of serum ATG5 level with CD4^+^ T-cell subset in stroke patients

**Figure 2:**
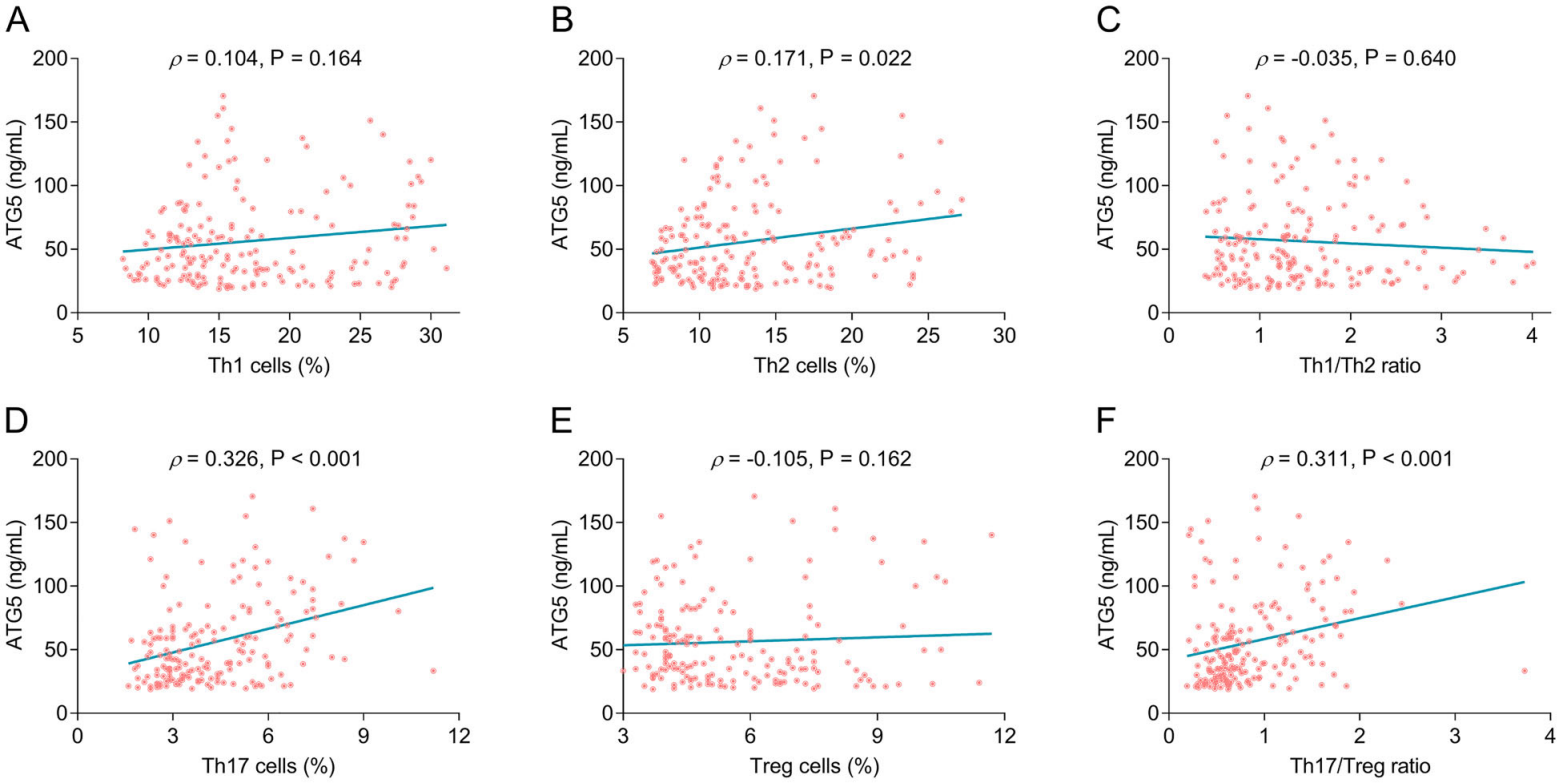
Serum autophagy-related gene 5 (ATG5) level was positively correlated to Th2 and Th17 cells in stroke patients. Spearman correlation between serum ATG5 level and Th1 cells (**A**), Th2 cells (**B**), Th1/Th2 ratio (**C**), Th17 cells (**D**), Treg cells (**E**), and Th17/Treg ratio (**F**) in stroke patients.

Serum ATG5 level was positively correlated to Th2 cells (P=0.022) in stroke patients but not Th1 cells (P=0.164) or Th1/Th2 ratio (P=0.640) (Figure 2A-C). Serum ATG5 level had a positive association with Th17 cells (P<0.001) and Th17/Treg ratio (P<0.001) in stroke patients, but not Treg cells (P=0.162) (Figure 2D-F).

### Comparison of serum ATG5 level and CD4^+^ T-cell subsets between stroke patients with and without cognition impairment

Serum ATG5 level was elevated in stroke patients with cognition impairment compared to those without (P=0.037). Th1 cells were elevated in patients with cognition impairment *vs* those without (P=0.022), whereas Th2 cells (P=0.611) and Th1/Th2 ratio (P=0.278) were not different between those patients. Th17 cells were increased in stroke patients with cognition impairment compared to those without (P=0.002), while Treg cells were not different between those patients (P=0.834). Th17/Treg ratio was increased in stroke patients with cognition impairment compared to those without (P=0.018) (Table 2).

**Table 2:** Comparisons of autophagy-related gene 5 (ATG5) level and CD4^+^ T-cell subset between patients with and without cognition impairment.

Items	Cognition impairment		*Z* value	P value
	No (n=101)	Yes (n=79)		
ATG5 (ng/mL)	42.1 (27.7-65.1)	54.4 (33.4-85.5)	-2.083	0.037
Th1 cells (%)	14.5 (12.4-19.8)	16.1 (13.5-22.6)	-2.289	0.022
Th2 cells (%)	11.6 (9.2-15.9)	12.1 (9.9-16.9)	-0.509	0.611
Th1/Th2 ratio	1.3 (0.8-1.8)	1.4 (0.9-2.0)	-1.085	0.278
Th17 cells (%)	3.4 (2.8-5.1)	4.8 (3.2-6.0)	-3.150	0.002
Treg cells (%)	5.1 (4.1-6.9)	4.9 (4.1-7.2)	-0.209	0.834
Th17/Treg ratio	0.7 (0.5-1.1)	0.8 (0.6-1.4)	-2.364	0.018

Th: T helper; Treg: regulatory T cells. Data are reported as median and interquartile range. Mann-Whitney U test.

### Correlation between serum ATG5 level and MMSE score in stroke patients

**Figure 3:**
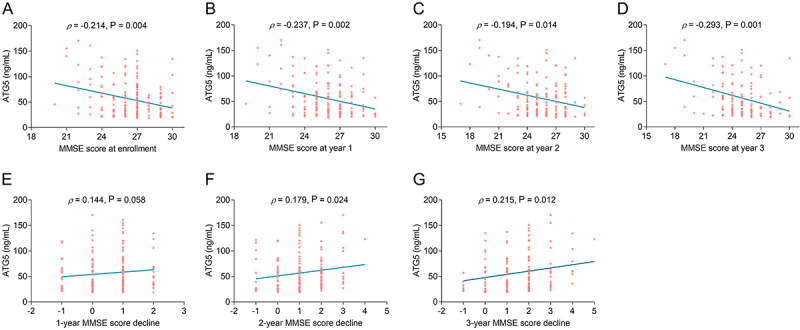
Elevated serum autophagy-related gene 5 (ATG5) level was associated with decreased Mini-Mental State Examination (MMSE) score and increased MMSE score decline in stroke patients. Spearman correlation between serum ATG5 level and MMSE score at enrollment (**A**), year 1 (**B**), year 2 (**C**), and year 3 (**D**), as well as 1-year (**E**), 2-year (**F**), and 3-year (**G**) MMSE score decline in stroke patients.

Serum ATG5 level was negatively correlated to MMSE score at enrollment (P=0.004) (Figure 3A), year 1 (P=0.002) (Figure 3B), year 2 (P=0.014) (Figure 3C), and year 3 (P=0.001) (Figure 3D) in stroke patients. Although serum ATG5 was not related to 1-year MMSE score decline (P=0.058) (Figure 3E), it was positively associated with 2-year (P=0.024) (Figure 3F) and 3-year (P=0.012) (Figure 3G) MMSE score decline in stroke patients.

### Correlation of CD4^+^ T-cell subset with MMSE score in stroke patients

Th1 cells were negatively correlated to MMSE score at enrollment (P=0.015), year 1 (P=0.001), and year 2 (P=0.032), but not at year 3 (P=0.140) in stroke patients. No association was found between Th2 cells or Th1/Th2 ratio and MMSE score at enrollment, year 1, year 2, or year 3 (all P>0.050). Decreased Th17 cells were associated with increased MMSE score at enrollment (P=0.001), year 1 (P<0.001), year 2 (P=0.001), and year 3 (P=0.004) in stroke patients. Th17/Treg ratio was negatively correlated with MMSE score at enrollment (P=0.013), year 1 (P=0.005), year 2 (P=0.026), and year 3 (P=0.022) in stroke patients, but no relationship between Treg cells and MMSE score at the aforementioned time points was found (all P>0.050) (Table 3).

**Table 3:** Spearman correlation of CD4^+^ T-cell subsets with MMSE score.

Items	MMSE score							
	At enrollment		At year 1		At year 2		At year 3	
	ρ value	P value	ρ value	P value	ρ value	P value	ρ value	P value
Th1 cells (%)	-0.180	0.015	-0.241	0.001	-0.170	0.032	-0.127	0.140
Th2 cells (%)	-0.109	0.145	-0.049	0.524	-0.071	0.377	-0.124	0.152
Th1/Th2 ratio	-0.036	0.629	-0.113	0.139	-0.062	0.437	0.009	0.913
Th17 cells (%)	-0.255	0.001	-0.275	<0.001	-0.252	0.001	-0.248	0.004
Treg cells (%)	-0.034	0.648	-0.011	0.887	-0.044	0.584	-0.008	0.926
Th17/Treg ratio	-0.185	0.013	-0.212	0.005	-0.177	0.026	-0.197	0.022

MMSE: mini-mental state examination; Th: T helper; Treg: regulatory T.

### Correlation of CD4^+^ T-cell subset with MMSE score decline in stroke patients

Th17 cells (P=0.037) and Th17/Treg ratio (P=0.036) were positively associated with MMSE score decline at year 1, but not at year 2 or year 3 (all P>0.050) in stroke patients. No correlation was found between Th1 cells, Th2 cells, Th1/Th2 ratio, or Treg cells and MMSE score declined at year 1, year 2, or year 3 (all P>0.050) in stroke patients (Table 4).

**Table 4:** Spearman correlation of CD4^+^ T-cell subsets with MMSE score decline.

Items	MMSE score decline					
	1-year		2-year		3-year	
	ρ value	P value	ρ value	P value	ρ value	P value
Th1 cells (%)	0.135	0.077	0.021	0.789	-0.003	0.977
Th2 cells (%)	-0.023	0.768	0.012	0.883	0.109	0.208
Th1/Th2 ratio	0.086	0.260	-0.002	0.978	-0.087	0.316
Th17 cells (%)	0.159	0.037	0.148	0.064	0.136	0.115
Treg cells (%)	-0.087	0.257	-0.044	0.582	-0.099	0.250
Th17/Treg ratio	0.160	0.036	0.135	0.091	0.154	0.074

MMSE: mini-mental state examination; Th: T helper; Treg: regulatory T.

## Discussion

This study found that: 1) elevated serum ATG5 level was correlated to elevated Th2 and Th17 cells in stroke patients; 2) increased Th17 cells was associated with worse cognition impairment in stroke patients; and 3) serum ATG5 level was positively correlated with incidence and deterioration of cognition impairment in stroke patients.

ATG5 regulates the formation of the autophagosome and is one of the most common regulators of autophagy (19,20). Autophagy is an intracellular conserved degradative process, involved in various physiological roles (cellular homeostasis, antigen presentation, protein quality control, etc.) (20,21). In addition, autophagy can mediate CD4^+^ T-cell differentiation via transcription factor or cytokine profiling (22,23). However, few studies have indicated the effect of ATG5 on CD4^+^ T-cell differentiation in stroke. This study found that elevated serum ATG5 level was related to elevated Th2 and Th17 cells in stroke patients. The possible reason could be that increased serum ATG5 induces autophagy and the latter facilitates T cells differentiating into Th2 and Th17 cells (22-24).

Th cells have been shown to reflect cognition impairment in some studies (25-27). For example, one study indicates that Th17 cells can estimate the progression of cognition impairment in stroke patients (25). Another study elucidates that elevated Th17 cells reflect a higher risk of cognition impairment (26). In addition, Th17 may be a potential biomarker for stroke cognition impairment prediction (27). This study found that increased Th17 cells were associated with worse cognition impairment in stroke patients. The possible reason could be that elevated Th17 cells promote pro-inflammatory cytokines, impair blood-brain barrier integrity, produce neuroinflammation, and induce neuronal apoptosis, leading to aggravated cognition impairment (25,27). As healthy controls were enrolled for comparing the expression of ATG5 with stroke patients, CD4^+^ T-cell subsets of healthy controls were not determined. However, considering that CD4^+^ T-cell subsets are likely to change with age (28), the change in CD4^+^ T-cell subset and its comparison between stroke patients and healthy controls as well as its correlation with cognition decline in healthy controls should be explored in the future.

As established in one study, overexpression of ATG5 affects neurogenesis and cognition function (29). In addition, other clinical studies indicate that serum ATG5 level is related to cognition impairment (30,31). For instance, a correlation of plasma ATG5 level with cognition impairment has been found in patients with Parkinson's disease (30). Another study elucidates that plasma ATG5 level is increased and positively correlated with cognition impairment in Alzheimer’s patients (31). This study found that serum ATG5 level was positively correlated with incidence and deterioration of cognition impairment in stroke patients. The possible reason could be that: 1) elevated serum ATG5 dysregulates autophagic flux, resulting in neuronal loss (31); 2) excessive serum ATG5 contributes to ferroptosis in brain IRI, thus promoting ischemic damage (12); 3) serum ATG5 regulates secretion of mature IL-1β and prevent its degradation (32), which is involved in vascular cognition impairment (33); and 4) according to the findings of this study, serum ATG5 level was positively correlated with Th17 cells, and Th17 cells might aggravate cognition impairment by promoting neural inflammation and apoptosis (22-25,27). Thus, it was assumed that the estimating value of ATG5 for cognition impairment was due to its positive correlation with Th17 cells. However, several studies have reported that the ATG gene family plays an important role in cognition impairment through multiple pathways [Supplementary Figure S1; (34-38)]. Thereby, further studies are required to validate that the correlation of ATG5 with Th2 and Th17 cells could impact cognitive decline in stroke patients by eliminating the influence of other ATGs on promoting cognition impairment.

Some limitations existed in the current study. To begin with, this study was a single-center study, which might result in selection bias. In addition, serum ATG5 level was detected at enrollment in stroke patients, while its long-term variation was unclear. Thirdly, this study did not explore other CD4^+^ T-cell subsets in stroke patients as well as their correlation with ATG5, such as Th9 and Th22 cells. Fourthly, the influence of APOε4 on the association of ATG5 with Th2 and Th17 cells in stoke patients remains unclear in this study. Finally, the detailed mechanism by which ATG5 regulated Th cell differentiation in stroke requires further investigation.

In conclusion, serum ATG5 level not only correlated with Th2 and Th17 cells, but also exhibited a predictive value for the incidence and deterioration of cognition impairment in stroke patients.

## Supplementary Material

Click to view [pdf].

## References

[1] Campbell BCV, Khatri P (2020). Stroke. Lancet.

[2] Collaborators GBDS (2021). Global, regional, and national burden of stroke and its risk factors, 1990-2019: a systematic analysis for the Global Burden of Disease Study 2019. Lancet Neurol.

[3] Rost NS, Brodtmann A, Pase MP, van Veluw SJ, Biffi A, Duering M (2022). Post-stroke cognitive impairment and dementia. Circ Res.

[4] Kalaria RN, Akinyemi R, Ihara M (2016). Stroke injury, cognitive impairment and vascular dementia. Biochim Biophys Acta.

[5] Huang YY, Chen SD, Leng XY, Kuo K, Wang ZT, Cui M (2022). Post-stroke cognitive impairment: epidemiology, risk factors, and management. J Alzheimers Dis.

[6] Lo Coco D, Lopez G, Corrao S (2016). Cognitive impairment and stroke in elderly patients. Vasc Health Risk Manag.

[7] Zhang X, Bi X (2020). Post-Stroke cognitive impairment: a review focusing on molecular biomarkers. J Mol Neurosci.

[8] Xu J, Xia L, Shang Q, Du J, Zhu D, Wang Y (2017). A variant of the autophagy-related 5 gene is associated with child cerebral palsy. Front Cell Neurosci.

[B09] Zhao H, Dong F, Li Y, Ren X, Xia Z, Wang Y (2021). Inhibiting ATG5 mediated autophagy to regulate endoplasmic reticulum stress and CD4(+) T lymphocyte differentiation: Mechanisms of acupuncture's effects on asthma. Biomed Pharmacother.

[B10] Pua HH, Dzhagalov I, Chuck M, Mizushima N, He YW (2007). A critical role for the autophagy gene Atg5 in T cell survival and proliferation. J Exp Med.

[B11] Razani B, Feng C, Coleman T, Emanuel R, Wen H, Hwang S (2012). Autophagy links inflammasomes to atherosclerotic progression. Cell Metab.

[B12] Zhu H, Zhong Y, Chen R, Wang L, Li Y, Jian Z (2022). ATG5 Knockdown attenuates ischemia-reperfusion injury by reducing excessive autophagy-induced ferroptosis. Transl Stroke Res.

[B13] Leiva-Rodrĺguez T, Romeo-Guitart D, Marmolejo-Martĺnez-Artesero S, Herrando-Grabulosa M, Bosch A, Fores J (2018). ATG5 overexpression is neuroprotective and attenuates cytoskeletal and vesicle-trafficking alterations in axotomized motoneurons. Cell Death Dis.

[B14] Zhou Y, Ge Y, Liu Q, Li YX, Chao X, Guan JJ (2021). LncRNA BACE1-AS promotes autophagy-mediated neuronal damage through the miR-214-3p/ATG5 signalling axis In Alzheimer's disease. Neuroscience.

[B15] Xi Y, Dhaliwal JS, Ceizar M, Vaculik M, Kumar KL, Lagace DC (2016). Knockout of Atg5 delays the maturation and reduces the survival of adult-generated neurons in the hippocampus. Cell Death Dis.

[16] Ajoolabady A, Shademan B, Avci CB, Nikanfar M, Nourazarian A, Laghousi D (2022). Diagnostic potential of autophagy-5 protein, apolipoprotein B-48, and oxidative stress markers in serum of patients with early-stage ischemic stroke. World Neurosurg.

[17] Powers WJ, Rabinstein AA, Ackerson T, Adeoye OM, Bambakidis NC, Becker K (2019). Guidelines for the early management of patients with acute ischemic stroke: 2019 update to the 2018 Guidelines for the early management of acute ischemic stroke: a Guideline for Healthcare Professionals From the American Heart Association/American Stroke Association. Stroke.

[18] Wang C, Huo H, Li J, Zhang W, Liu C, Jin B (2022). The longitudinal changes of serum JKAP and IL-17A, and their linkage with anxiety, depression, and cognitive impairment in acute ischemic stroke patients. J Clin Lab Anal.

[19] Ye X, Zhou XJ, Zhang H (2018). Exploring the role of autophagy-related gene 5 (ATG5) Yields important insights into autophagy in autoimmune/autoinflammatory diseases. Front Immunol.

[20] Changotra H, Kaur S, Yadav SS, Gupta GL, Parkash J, Duseja A (2022). ATG5: a central autophagy regulator implicated in various human diseases. Cell Biochem Funct.

[21] Chinchwadkar S, Padmanabhan S, Mishra P, Singh S, Suresh SN, Vats S (2017). Multifaceted housekeeping functions of autophagy. J Indian Instit Sci.

[22] Jeong J, Choi YJ, Lee HK (2022). The role of autophagy in the function of CD4(+) T cells and the development of chronic inflammatory diseases. Front Pharmacol.

[B23] Wang L, Das JK, Kumar A, Peng HY, Ren Y, Xiong X (2021). Autophagy in T-cell differentiation, survival and memory. Immunol Cell Biol.

[B24] Park MJ, Lee SY, Moon SJ, Son HJ, Lee SH, Kim EK (2016). Metformin attenuates graft-*versus*-host disease via restricting mammalian target of rapamycin/signal transducer and activator of transcription 3 and promoting adenosine monophosphate-activated protein kinase-autophagy for the balance between T helper 17 and Tregs. Transl Res.

[25] Lu T, Ma L, Xu Q, Wang X (2022). Blood Th17 cells and IL-17A as candidate biomarkers estimating the progression of cognitive impairment in stroke patients. J Clin Lab Anal.

[B26] Zhou Y, Yu K (2022). Th1, Th2, and Th17 cells and their corresponding cytokines are associated with anxiety, depression, and cognitive impairment in elderly gastric cancer patients. Front Surg.

[27] Yu S, Cui W, Han J, Chen J, Tao W (2022). Longitudinal change of Th1, Th2, and Th17 cells and their relationship between cognitive impairment, stroke recurrence, and mortality among acute ischemic stroke patients. J Clin Lab Anal.

[28] Zhou L, Leonard A, Pavel AB, Malik K, Raja A, Glickman J (2019). Age-specific changes in the molecular phenotype of patients with moderate-to-severe atopic dermatitis. J Allergy Clin Immunol.

[29] Vázquez P, Arroba AI, Cecconi F, de la Rosa EJ, Boya P, de Pablo F (2012). Atg5 and Ambra1 differentially modulate neurogenesis in neural stem cells. Autophagy.

[30] Han J, Feng G, Wu J, Zhang Y, Long Z, Yao X (2022). Association of ATG5 gene polymorphism with Parkinson's disease in a Han Chinese population. Acta Neurol Belg.

[31] Cho SJ, Lim HJ, Jo C, Park MH, Han C, Koh YH (2019). Plasma ATG5 is increased in Alzheimer's disease. Sci Rep.

[32] Dupont N, Jiang S, Pilli M, Ornatowski W, Bhattacharya D, Deretic V (2011). Autophagy-based unconventional secretory pathway for extracellular delivery of IL-1beta. EMBO J.

[33] Poh L, Sim WL, Jo DG, Dinh QN, Drummond GR, Sobey CG (2022). The role of inflammasomes in vascular cognitive impairment. Mol Neurodegener.

[34] Hara T, Nakamura K, Matsui M, Yamamoto A, Nakahara Y, Suzuki-Migishima R (2006). Suppression of basal autophagy in neural cells causes neurodegenerative disease in mice. Nature.

[B35] Xie C, Ginet V, Sun Y, Koike M, Zhou K, Li T (2016). Neuroprotection by selective neuronal deletion of Atg7 in neonatal brain injury. Autophagy.

[B36] Choi I, Zhang Y, Seegobin SP, Pruvost M, Wang Q, Purtell K (2020). Microglia clear neuron-released alpha-synuclein via selective autophagy and prevent neurodegeneration. Nat Commun.

[B37] Li Y, Zhang Y, Wang L, Wang P, Xue Y, Li X (2017). Autophagy impairment mediated by S-nitrosation of ATG4B leads to neurotoxicity in response to hyperglycemia. Autophagy.

[38] Tamargo-Gómez I, Martínez-García GG, Suárez MF, Rey V, Fueyo A, Codina-Martínez H (2021). ATG4D is the main ATG8 delipidating enzyme in mammalian cells and protects against cerebellar neurodegeneration. Cell Death Differ.

