# Supplementary material for: Serum autophagy-related gene 5 level in stroke patients: correlation with CD4+ T cells and cognition impairment during a 3-year follow-up
Source: Braz J Med Biol Res. 2024 Mar 18;57:e13019. doi: 10.1590/1414-431X2024e13019 (PMC10946239; doi:10.1590/1414-431X2024e13019)
Supplement: Supplementary file 1 [file 1414-431X-bjmbr-57-e13019-suppl.pdf]

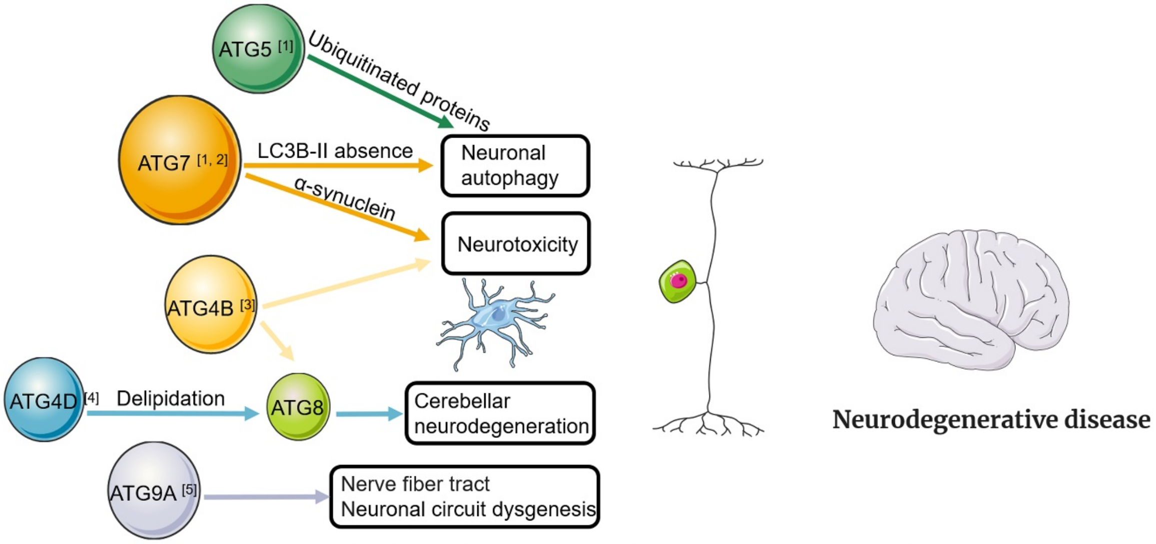


1. Hara T, Nakamura K, Matsui M, Yamamoto A, Nakahara Y, Suzuki-Migishima R, et al. Suppression of basal autophagy in neural cells causes neurodegenerative disease in mice. *Nature* 2006; 441: 885-9, doi: 10.1038/nature04724.
2. Xie C, Ginet V, Sun Y, Koike M, Zhou K, Li T, et al. Neuroprotection by selective neuronal deletion of Atg7 in neonatal brain injury. *Autophagy* 2016; 12: 410-23, doi: 10.1080/15548627.2015.1132134.
3. Choi I, Zhang Y, Seegobin SP, Pruvost M, Wang Q, Purtell K, et al. Microglia clear neuron-released α-synuclein via selective autophagy and prevent neurodegeneration. *Nat Commun* 2020; 11: 1386, doi: 10.1038/s41467-020-15119-w.
4. Li Y, Zhang Y, Wang L, Wang P, Xue Y, Li X, et al. Autophagy impairment mediated by S-nitrosation of ATG4B leads to neurotoxicity in response to hyperglycemia. *Autophagy* 2017; 13: 1145-1160, doi: 10.1080/15548627.2017.1320467.
5. Tamargo-Gómez I, Martínez-García GG, Suárez MF, Rey V, Fueyo A, Codina-Martínez H, et al. ATG4D is the main ATG8 delipidating enzyme in mammalian cells and protects against cerebellar neurodegeneration. *Cell Death Differ* 2021; 28: 2651-2672, doi: 10.1038/s41418-021-00776-1.

**Figure S1.** Autophagy-related gene (ATG) family engages in neurodegenerative disease via multiple pathways.

**Table S1.** $CD4^+$ T-cell subsets in males and females.

| Items | Gender | | *Z* value | P value |
|---|---|---|---|---|
| | Females (n=66) | Males (n=114) | | |
| Th1 cells (%) | 15.8 (12.9–20.8) | 15.3 (12.6–20.7) | –0.205 | 0.838 |
| Th2 cells (%) | 11.8 (9.5–15.6) | 12.3 (9.7–17.7) | –0.466 | 0.641 |
| Th1/Th2 ratio | 1.3 (1.0–1.9) | 1.3 (0.8–1.9) | –0.332 | 0.740 |
| Th17 cells (%) | 4.0 (2.9–5.8) | 3.9 (2.9–5.3) | –0.576 | 0.565 |
| Treg cells (%) | 5.3 (4.2–7.3) | 4.9 (4.1–6.8) | –1.027 | 0.304 |
| Th17/Treg ratio | 0.7 (0.5–1.2) | 0.7 (0.5–1.2) | –0.045 | 0.964 |

Th: T helper; IQR: interquartile range; Treg: regulatory T. Data are reported as median and interquartile range. Mann-Whitney U test.
